# Metabolic changes in children with idiopathic central precocious puberty after gonadotrophin-releasing hormone agonist therapy: a meta-analysis

**DOI:** 10.3389/fped.2025.1519746

**Published:** 2025-08-29

**Authors:** Bo Zhou, Xia Qu, Jianhong Wang, Qi Xu, Lili Zhang, Xi Wang, Nan Peng, Jialu Gu, Xiaoqian Zhang, Qiong Wang, Wenquan Niu, Lin Wang

**Affiliations:** ^1^ Child Healthcare Center, Capital Center for Children’s Health, Capital Medical University, Beijing, China; ^2^ Graduate School, Beijing University of Chinese Medicine, Beijing, China; ^3^ Center for Evidence-Based Medicine, Capital Institute of Pediatrics, Beijing, China

**Keywords:** gonadotrophin-releasing hormone agonists, children, idiopathic central precocious puberty, metabolic changes, meta-analysis

## Abstract

**Background:**

We aimed to assess the effects of gonadotrophin-releasing hormone agonist (GnRHa) therapy on metabolic changes by synthesizing results from clinical trials involving children with idiopathic central precocious puberty (ICPP).

**Methods:**

Literature search, trial selection, data extraction and quality assessment were completed independently by two investigators. STATA software (version 14.1) was used for data analyses. Effect-size estimates are expressed as weighted mean difference (WMD) with 95% confidence interval (CI).

**Results:**

This meta-analysis was conducted based on 19 clinical trials and 1,553 ICPP children. Overall analyses showed that for body mass index standard deviation score (BMISDS), no significance was noted after administering GnRHa to children with ICPP (WMD: −0.08; 95% CI: −0.22–0.06; *p* = 0.269). Similarly, no significance was noted for total cholesterol (WMD: 1.94 mg/dl; 95% CI: −10.29–14.17; *p* = 0.756), triglyceride (WMD: −5.31 mg/dl; 95% CI: −26.92–16.29; *p* = 0.630) and low-density lipoprotein (WMD: −9.63 mg/dl; 95% CI: −40.09–20.83; *p* = 0.535). By contrast, a statistically higher high-density lipoprotein of 7.07 mg/dl after administering GnRHa to children with ICPP (95% CI: 3.00–11.14; *p* = 0.001). Subgroup and meta-regression analyses revealed that initial body weight, sample size, and age were significant sources of between-trial heterogeneity. There was a low probability of publication bias for above comparison, as indicated by Egger's tests.

**Conclusions:**

Our meta-analytical findings indicate that GnRHa treatment did not appear to increase BMI and lipid metabolism levels in children with ICPP, irrespective of obesity status at the time of initiation therapy.

**Systematic Review Registration:**

PROSPERO (CRD42023410554).

## Introduction

Precocious puberty is defined as the appearance of secondary sexual characteristics before the age of 8 years in girls and 9 years in boys. Precocious puberty can be divided into central precocious puberty (CPP) and peripheral precocious puberty (PPP), and most of them are idiopathic central precocious puberty (ICPP). In the Danish study, the prevalence of precocious puberty was 0.2% for girls and <0.05% for boys (1). Boys with precocious puberty are less common, but more likely to reflect serious diseases (2). In a longitude study from Korea, the prevalence of central precocious puberty was 0.56% in girls and 0.0017% in boys, and the annual incidence has substantially increased over past 7 years (3). Besides loss of final adult height, children with ICPP may be at higher risk for other consequences including behavioral problems, breast cancer, obesity, and metabolic comorbidities (4), arousing wide public concerns worldwide. Therefore, effective treatment targeting ICPP is urgently needed to curb these unexpected consequences.

Some ICPP children can benefit from the treatment of GnRH analogues (GnRHa) to preserve final adult height potential, postpone menarcheal age, and alleviate negative psychosocial stress (5). Sustained high concentrations of GnRHa make the GnRH receptor occupied and hypothalamic-pituitary-gonadal axis is subsequently suppressed, inhibiting gonadal steroid secretion and return of sex steroids to prepubertal levels (6). It is widely accepted that obesity, family history and gene susceptibility are associated with ICPP in children (7). Several longitudinal and cross-sectional studies have shown a significant correlation between female obesity and earlier pubertal development (8–12). In the last decades, it is demonstrated that GnRHa is generally safe. However, metabolic abnormalities associated with GnRHa treatment on CPP remain controversial (13). Some studies have shown that GnRHa treatment may be associated with obesity, increased insulin resistance, hyperandrogenemia, and polycystic ovary syndrome. Corripio et al. (14) found a significant increase in BMISDS in girls receiving GnRHa treatment, with persistent increase until the therapy was stopped and adult height was achieved. While Wolters et al. (15) and Aguiar et al. (16) reported that the increase of BMI was mainly in girls with normal weight before treatment. A cohort of 46 girls using long-acting goserelin indicated that overweight girls were at higher risk to become fatter after GnRHa treatment (17). In a Korea study, there was an increase in body mass index (BMI) z-scores after treatment with GnRHa in girls with CPP; however, changes in insulin resistance were not evaluated (18, 19). On the contrary, several studies showed that GnRHa treatment had no effects on BMI and obesity rates (20, 21), although CPP was associated with obesity. Moreover, Colmenares et al. (21) reported that the fasting glucose and lipids profiles remained in the normal range and stable during follow-ups. To shed some light on these controversial reports, a comprehensive assessment on this subject is needed, yet currently lacking.

For this meta-analysis, we hypothesized that GnRHa administration can impact metabolic changes of children with ICPP and weight at the beginning of treatment can contribute to the weight gain after the therapy. A comprehensive meta-analysis of comparative controlled trials on metabolic changes after administering GnRHa to children with ICPP will be used to test our hypothesis.

## Methods

We conducted this meta-analysis in accordance with the Preferred Reporting Items for Systematic Reviews and Meta-Analyses (PRISMA) reporting guidelines. The PRISMA checklist is provided in Supplementary Table 1. This meta-analysis was registered on the International Prospective Register of Systematic Reviews (PROSPERO) (CRD42023410554).

### Search strategy

A literature search of the PubMed, EMBASE (Excerpt Medica Database), Cochrane Library, and Web of Science databases was conducted up to October 15, 2024 for comparative controlled trials on GnRHa and precocious puberty. The Medical Subject Headings (MeSH) were used for literature search, and they are expressed in the Boolean format: (GnRHa OR LHRHa OR Triptorelin OR Decapeptyl OR Leuprorelin OR Enanton OR Buserelin OR Deslorelin OR Gonadorelin OR gonadotropin-releasing hormone analogue OR Gonadotropin-Releasing Hormone/analogs OR Gonadotropin-Releasing Hormone OR gonadotropin-releasing hormone OR GnRH analogues OR Goserelin OR Triptorelin Pamoate OR Leuprolide) AND (Puberty, Precocious OR Precocious Puberties OR Puberties, Precocious OR Pubertas Praecox OR Praecox, Pubertas OR Precocious Puberty OR Precocious Puberty, Central OR Central Precocious Puberties OR Central Precocious Puberty OR Precocious Puberties, Central OR Puberties, Central Precocious OR Puberty, Central Precocious OR Sexual Precocity OR Precocities, Sexual OR Precocity, Sexual OR Sexual Precocities OR Idiopathic Sexual Precocity OR Idiopathic Sexual Precocities OR Precocities, Idiopathic Sexual OR Precocity, Idiopathic Sexual OR Sexual Precocities, Idiopathic OR Sexual Precocity, Idiopathic OR Familial Precocious Puberty OR Familial Precocious Puberties OR Precocious Puberties, Familial OR Precocious Puberty, Familial OR Puberties, Familial Precocious OR Puberty, Familial Precocious OR Precocious Puberty, Male-Limited OR Male-Limited Precocious Puberties OR Male-Limited Precocious Puberty OR Precocious Puberties, Male-Limited OR Puberties, Male-Limited Precocious OR Puberty, Male-Limited Precocious OR Precocious Puberty, Male Limited OR Testotoxicosis). Reference lists of relevant reviews and original articles were searched manually to ensure no missing hits. Two authors (X.Q. and J.W.) completed the literature search independently, and discussion was conducted with a third investigator (B.Z.) when there was any divergence.

### Eligibility criteria

Retrieved articles meeting the following inclusion criteria were included: (i) study type: prospective or retrospective clinical control trials; (ii) participants: children with ICPP; (iii) intervention: GnRHa treatment; (iv) available BMI; (v) language of publication: English. ICPP was diagnosed in accordance with the following criteria (5, 22): (1) breast development or other secondary sex characteristics such as pubic hair and axillary hair growth before the CA of 8 years or menarche before the CA of 10 years in girls, testicular volume ≥4 ml and genitalia ≥Tanner stage 2 before the chronological age (CA) of 9 years in boys; (2) advanced bone ages (BA) ≥1 year above the CA; (3) LH peak values ≥5 IU/L at the GnRH stimulation test; (4) a normal brain magnetic resonance imaging with a thorough examination of the hypothalamic pituitary region**.**

### Data extraction

Two investigators (X.Q. and B.Z.) independently extracted the qualitative and quantitative data from qualified articles into a predesigned template, including (i) methodological characteristics: name of the first author, country where participants were enrolled, year of publication, sample size of each group, study type, the initial body weight; (ii) demographic characteristics: gender composition, baseline age, baseline bone age (BA), intervention in both arms, duration of therapy; (iii) clinical outcomes. The primary outcome is the difference of BMI standard deviation score (BMISDS) in both groups, which is calculated as (measured BMI—mean BMI)/standard deviation (SD). Measured BMI is the actual BMI of a child, and mean BMI and SD are mean BMI and standard deviation for children of a corresponding age. The secondary outcomes include the difference of triglyceride (TG), total cholesterol (TC), low-density lipoprotein (LDL), and high-density lipoprotein (HDL). Kappa statistic was used to compare for consistency of the extracted data. Any disagreements between the two investigators were resolved by a third investigator (W.N.).

### Quality assessment

Non-randomized controlled trials were assessed using the Methodological Index of Non-randomized Studies (MINORS) scoring system (23). It has 12 indicators, each of which was scored from 0 to 2 points, with the total score ranging from 0 (the worst) to 24 (the best).

### Statistical analyses

Quantitative outcomes changing from baseline to endline were compared and expressed as weighted mean difference (WMD) and 95% confidence interval (CI). The random-effects model using the Der Simonian and Laird method (24) was used to pool individual trials. Heterogeneity was tested by the *χ^2^* test with *p* < 10% suggesting statistically significant, and it was measured by inconsistency index (*I^2^*) statistic with *I^2^* > 50% suggesting statistically significant. Subgroup analysis and meta-regression analysis were done to explore potential causes of between-trial heterogeneity.

Cumulative analysis was conducted to evaluate the effects of the first published trial on subsequent trials and the evolution of accumulated estimates over time. Influential analysis was employed to assess the contribution of individual trials to the overall estimate.

Publication bias was justified by using the Begg's funnel plots and Egger's tests at a significance level of 10% (25). The potentially missing trials were estimated using the trim-and-fill method. A probability of <0.05 was considered to indicate statistical significance. All data were analyzed by the STATA software special edition (Stata Corp, College Station, TX, version 14.1 for Windows).

## Results

### Eligible trials

**Figure 1:**
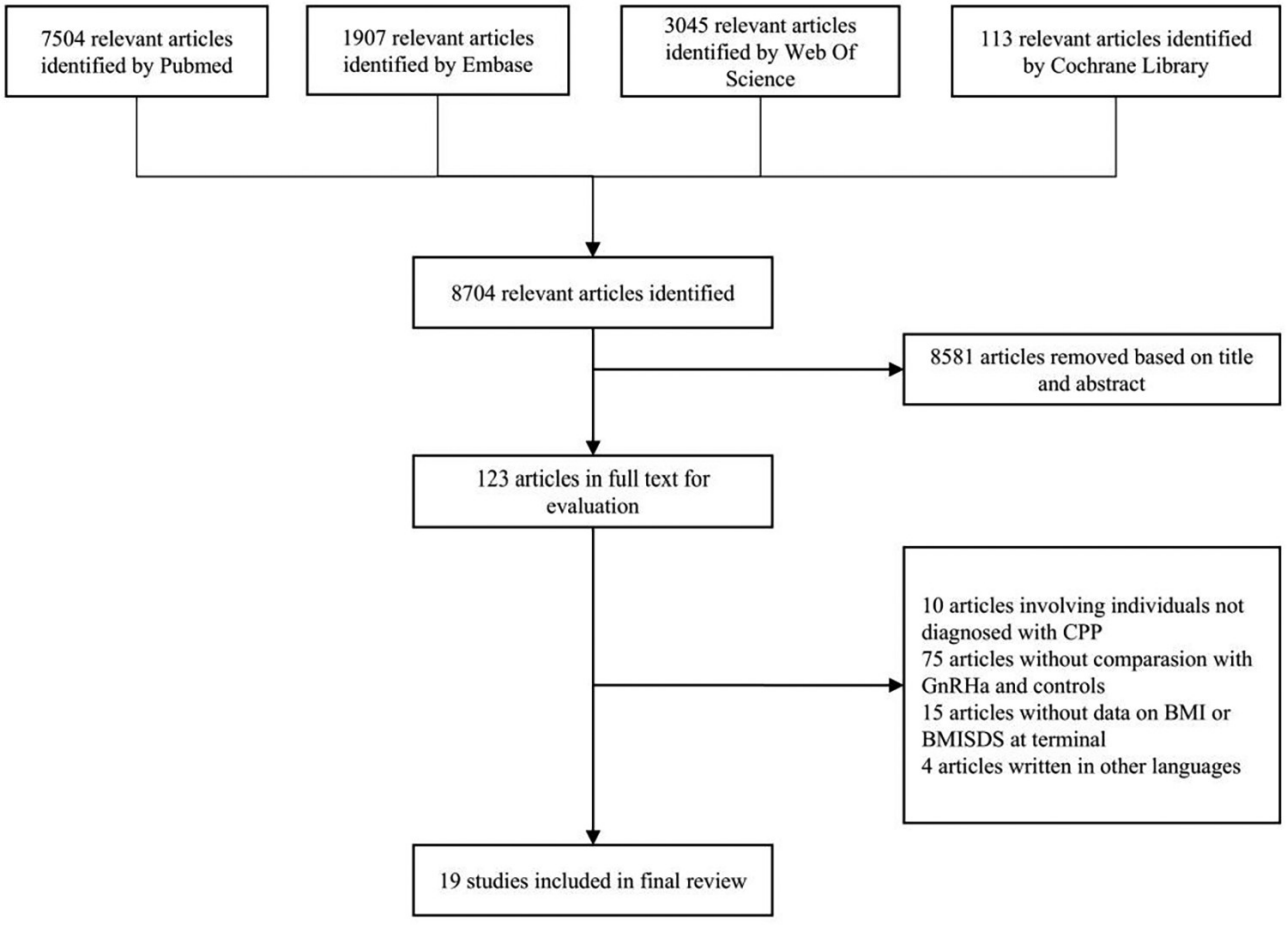
Flow diagram illustrating the selection of qualified clinical trials with specific reasons for exclusion. GnRHa, gonadotrophin-releasing hormone agonists; CPP, central precocious puberty; BMI, body mass index; SDS, standard deviation score.

A total of 8,704 relevant publications were initially identified after literature search. Based on titles and abstracts, 8,581 articles were excluded with obvious reasons, leaving 123 articles for further evaluation in full texts. Finally, there were 19 clinical trials (15, 20, 21, 26–41) including 1,553 children for analysis, and all trials were published from the year 2000 to 2024. In the case of some trials from the same study population, the trial published with the most complete information was selected. Figure 1 presents the selection process of eligible publications with specific reasons for exclusion.

### Characteristics of eligible trials

The characteristics of all qualified trials are shown in Table 1. There were nine trials included children of all weights (20, 21, 26, 28–30, 35, 38, 41) and ten trials included children of overweight or obese (15, 27, 31–34, 36, 37, 39, 40). There were 12 trials compared with GnRHa treatment (15, 28–34, 36, 37, 39, 40) and seven trials compared with no treatment (20, 21, 26, 27, 35, 38, 41). Mean age ranged from 5.0 years to 9.43 years.

**Table 1:** Characteristics of qualiﬁed trials in this meta-analysis.

Author	Year	Continent	Study type	Sample size	The initial body weight	Control group	Age (years)	Bone age (years)	Initial BMISDS	
									Case group	Control group
Partsch (30)	2000	Europe	CCT	29/23	All weight	GnRHa	5/7	8.4/10.4	1.9 ± 0.29	2 ± 0.51
Lazar (29)	2007	Asia	CCT	60/55	All weight	GnRHa	6.12/8.5	8.67/10.8	0.6 ± 1	0.5 ± 0.6
Poomthavorn (41)	2011	Asia	CCT	47/11	All weight	Untreated	8.3/8.6	NA	1.26 ± 0.95	1.18 ± 1.33
Wolters (15)	2012	Europe	CCT	29/63	Overweight or obese	GnRHa	7.1/7.1	NA	2.01 ± 0.69	0.08 ± 1.02
Colmenares (21)	2014	South America	CCT	29/8	All weight	Untreated	7.3/7.7	NA	1.2 ± 0.9	1 ± 0.8
Arani (20)	2015	Asia	CCT	46/64	All weight	Untreated	7.89/7.16	9.66/8.43	0.84 ± 1.01	1.49 ± 1.28
Arcari (36)	2016	South America	CCT	32/28	Overweight or obese	GnRHa	NA	NA	1.7 ± 0.5	0.25 ± 0.8
Faienza (35)	2017	Europe	CCT	56/38	All weight	Untreated	7/7	10.1/10.2	0.4 ± 0.8	0.45 ± 0.9
Yang (40)	2017	Asia	CCT	31/46	Overweight or obese	GnRHa	8.4/8.6	10.4/10.3	1.7 ± 0.4	0.2 ± 0.7
Kim (37)	2017	Asia	CCT	32/97	Overweight or obese	GnRHa	NA	NA	1.89 ± 0.62	0 ± 0.71
Park (39)	2018	Asia	CCT	21/38	Overweight or obese	GnRHa	8.22/8.16	10.4/10.2	1.5 ± 0.39	−0.04 ± 0.72
Satitpatanapan (26)	2020	Asia	CCT	41/26	All weight	Untreated	7/8.4	10.5/13.3	0.69 ± 0.53	1.09 ± 0.45
Onat (28)	2020	Asia	CCT	19/35	All weight	GnRHa	7.26/8.97	NA	0.38 ± 0.83	0.72 ± 0.76
Lim (31)	2020	Asia	CCT	38/37	Overweight or obese	GnRHa	9.43/9.6	NA	1.57 ± 0.37	0.32 ± 0.4
Vuralli (34)	2020	Asia	CCT	56/82	Overweight or obese	GnRHa	8.4/8.6	10.6/10.7	1.66 ± 0.48	0.42 ± 0.54
Loochi (27)	2021	Asia	CCT	9/23	Overweight or obese	Untreated	8.9/8.9	NA	1.35 ± 0.06	0.24 ± 0.17
Donbaloğlu (32)	2022	Asia	CCT	17/26	Overweight or obese	GnRHa	7.39/NA	NA	1.39 ± 0.99	0.32 ± 1.44
Bruzzi (33)	2022	South America	CCT	24/33	Overweight or obese	GnRHa	7.06/7.86	9.33/9.58	1.48 ± 0.36	−0.01 ± 0.69
Tseng (38)	2023	Asia	CCT	109/95	All weight	Untreated	8.7/8.4	11.5/10.8	0.4 ± 1.2	0.1 ± 1.6

Author	Terminal BMISDS		Initial TC (mg/dl)		Terminal TC (mg/dl)		Initial TG (mg/dl)	
	Case group	Control group	Case group	Control group	Case group	Control group	Case group	Control group
Partsch (30)	2.4 ± 0.4	1.9 ± 0.37	NA	NA	NA	NA	NA	NA
Lazar (29)	0.59 ± 0.65	0.6 ± 0.6	NA	NA	NA	NA	NA	NA
Poomthavorn (41)	0.16 ± 1	0.59 ± 0.93	NA	NA	NA	NA	NA	NA
Wolters (15)	2.03 ± 0.54	0.4 ± 0.85	NA	NA	NA	NA	NA	NA
Colmenares (21)	1.3 ± 0.8	0.7 ± 0.5	156 ± 31.5	167.6 ± 35.7	159.2 ± 16.5	171.7 ± 39.1	83.3 ± 36.2	115.8 ± 78.5
Arani (20)	0.92 ± 0.82	1.39 ± 1.16	NA	NA	NA	NA	NA	NA
Arcari (36)	1 ± 0.9	−0.19 ± 0.7	NA	NA	NA	NA	NA	NA
Faienza (35)	0.35 ± 0.8	0.3 ± 0.9	NA	NA	158.8 ± 23.5	144 ± 32.6	NA	NA
Yang (40)	1.6 ± 0.52	0.39 ± 0.67	NA	NA	NA	NA	NA	NA
Kim (37)	1.92 ± 0.68	0.51 ± 0.77	NA	NA	NA	NA	NA	NA
Park (39)	1.51 ± 0.5	0.29 ± 0.65	NA	NA	NA	NA	NA	NA
Satitpatanapan (26)	1.12 ± 0.53	1.58 ± 0.45	NA	NA	181 ± 34	203 ± 45	NA	NA
Onat (28)	0.79 ± 0.72	0.84 ± 0.8	NA	NA	NA	NA	NA	NA
Lim (31)	1.53 ± 0.49	0.41 ± 0.46	NA	NA	NA	NA	NA	NA
Vuralli (34)	1.69 ± 0.53	0.87 ± 0.33	NA	NA	NA	NA	NA	NA
Loochi (27)	1.56 ± 0.09	0.42 ± 0.18	NA	NA	NA	NA	NA	NA
Donbaloğlu (32)	1.81 ± 1.97	0.46 ± 1.77	NA	NA	NA	NA	NA	NA
Bruzzi (33)	1.45 ± 0.28	0.28 ± 0.84	146.4 ± 18.8	157.41 ± 26.9	154.5 ± 22.69	162.91 ± 35.9	70 ± 20.37	65.86 ± 27.38
Tseng (38)	0.1 ± 1.275	−0.4 ± 1.6	NA	NA	NA	NA	NA	NA

Author	Terminal TG (mg/dl)		Initial LDL (mg/dl)		Terminal LDL (mg/dl)		Initial HDL (mg/dl)		Terminal HDL (mg/dl)	
	Case group	Control group	Case group	Control group	Case group	Control group	Case group	Control group	Case group	Control group
Partsch (30)	NA	NA	NA	NA	NA	NA	NA	NA	NA	NA
Lazar (29)	NA	NA	NA	NA	NA	NA	NA	NA	NA	NA
Poomthavorn (41)	NA	NA	NA	NA	NA	NA	NA	NA	NA	NA
Wolters (15)	NA	NA	NA	NA	NA	NA	NA	NA	NA	NA
Colmenares (21)	83.3 ± 52.7	98.5 ± 37.5	96.6 ± 38.1	99.5 ± 16.3	100.2 ± 22.8	128.8 ± 13.9	43.8 ± 9	52 ± 7.1	43.8 ± 14	44.5 ± 2.1
Arani (20)	NA	NA	NA	NA	NA	NA	NA	NA	NA	NA
Arcari (36)	NA	NA	NA	NA	NA	NA	NA	NA	NA	NA
Faienza (35)	59.4 ± 14.5	61.8 ± 24	NA	NA	87.2 ± 23.5	85.1 ± 20.5	NA	NA	56.7 ± 9.2	47.6 ± 2.5
Yang (40)	NA	NA	NA	NA	NA	NA	NA	NA	NA	NA
Kim (37)	NA	NA	NA	NA	NA	NA	NA	NA	NA	NA
Park (39)	NA	NA	NA	NA	NA	NA	NA	NA	NA	NA
Satitpatanapan (26)	91 ± 41	105 ± 49	NA	NA	125 ± 37	148 ± 45	NA	NA	58 ± 18	57 ± 12
Onat (28)	NA	NA	NA	NA	NA	NA	NA	NA	NA	NA
Lim (31)	NA	NA	NA	NA	NA	NA	NA	NA	NA	NA
Vuralli (34)	NA	NA	NA	NA	NA	NA	NA	NA	NA	NA
Loochi (27)	NA	NA	NA	NA	NA	NA	NA	NA	NA	NA
Donbaloğlu (32)	NA	NA	NA	NA	NA	NA	NA	NA	NA	NA
Bruzzi (33)	63.25 ± 36.2	69.8 ± 30.1	78.9 ± 16.8	85.8 ± 23.4	85 ± 20.63	86.5 ± 22.07	56.27 ± 6.2	59.2 ± 15.2	54.75 ± 11.9	51.09 ± 11.1
Tseng (38)	NA	NA	NA	NA	NA	NA	NA	NA	NA	NA

Data are expressed as case group/control group. GnRHa, gonadotrophin-releasing hormone agonists; CCT, clinical controlled trial; BMI SDS, body mass index standard deviation score; TC, total cholesterol; TG, triglyceride; LDL low-density lipoprotein; HDL, high-density lipoprotein; NA, not available.

### Quality assessment

According to the MINORS scoring system, total scores of all eligible trials ranged from 15 to 18 (mean: 16.42; SD: 0.86) (Supplementary Table 2).

### Overall analyses

**Figure 2:**
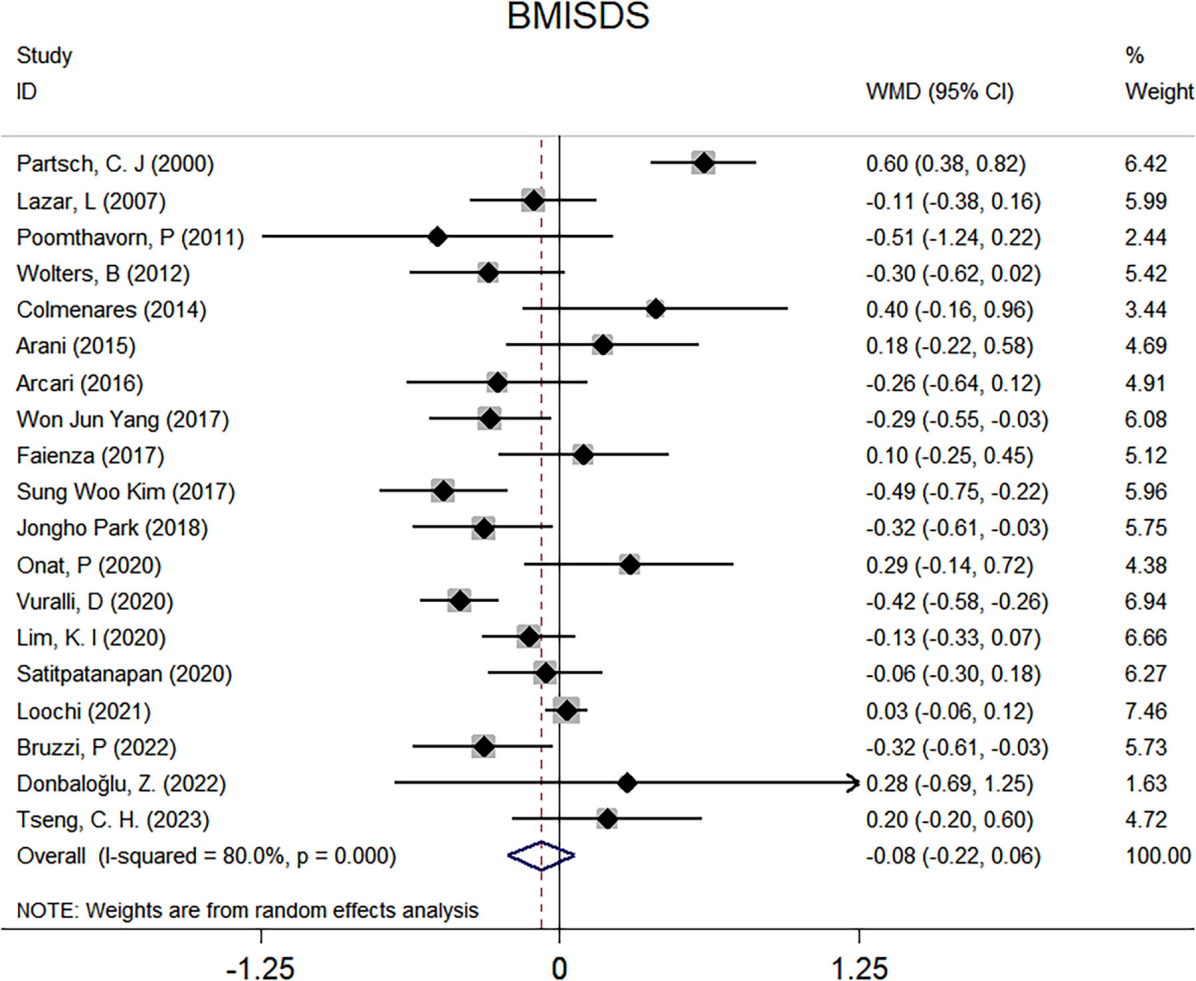
Forest plots for the comparison of BMISDS in overall analyses. BMI, body mass index; SDS, standard deviation score; WMD, weighted mean difference; 95% CI, 95% confidence interval.

Overall effect-size estimates for the difference of BMISDS, TC, TG, LDL, and HDL between intervention and control groups are provided in Figure 2 and Supplementary Figure 1.

There were 19 clinical trials for BMISDS, and no significance was noted after administering GnRHa to children with ICPP (WMD: −0.08; 95% CI: −0.22–0.06; *p* = 0.269), with high evidence of heterogeneity (*I*^2^: 80.0%; *p* < 0.001) (Figure 2). Two trials are available for TC, which revealed no significant differences after GnRHa treatment (WMD: 1.94 mg/dl; 95% CI: −10.29–14.17; *p* = 0.756), with no evidence of heterogeneity between trials (*I*^2^: 0.0%; *p* = 0.821) (Supplementary Figure 1A). Similarly, no significance was noted for TG (WMD: −5.31 mg/dl; 95% CI: −26.92–16.29; *p* = 0.630) and LDL (WMD: −9.63 mg/dl; 95% CI: −40.09–20.83; *p* = 0.535), with low and high evidence of heterogeneity (*I*^2^: 22.8% and 90.4%; *p* = 0.255 and 0.001) (Supplementary Figures 1B,C). By contrast, analysis of two trials revealed a statistically higher HDL of 7.07 mg/dl after administering GnRHa to children with ICPP (95% CI: 3.00–11.14; *p* = 0.001), and there was no evidence of heterogeneity between trials (*I*^2^: 0.0%; *p* = 0.827) (Supplementary Figure 1D).

### Subgroup analyses

**Figure 3:**
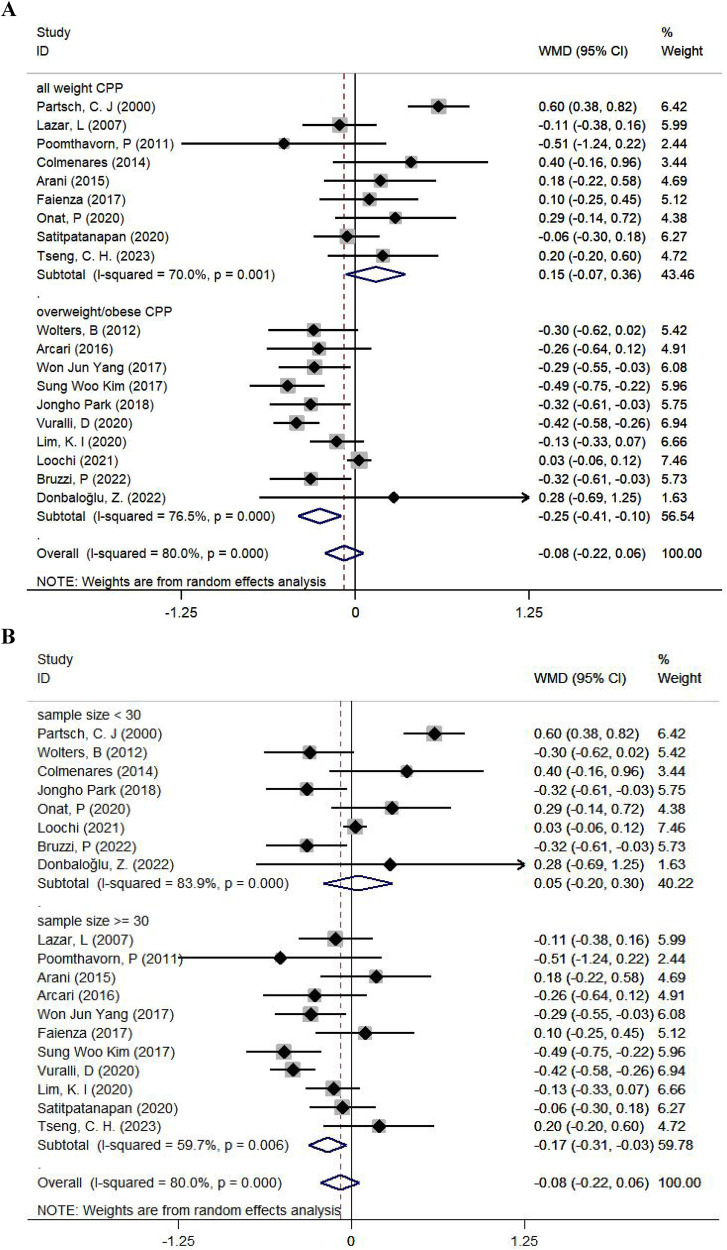
Subgroup analyses for the comparison of BMISDS according to the body weight at the initiation of treatment **(A)** and sample size **(B)**. CPP, central precocious puberty; WMD, weighted mean difference; 95% CI, 95% confidence interval.

Given the limited number of available trials for some comparison, subgroup analyses were done for the comparison of BMISDS separately by initial body weight, sample size, age, geographical region, treatment duration, and GnRHa usage status, as presented in Figure 3 and Supplementary Figure 2.

According to initial body weight, BMISDS was significantly decreased after administering GnRHa to children with ICPP and overweight or obesity (WMD: −0.25; 95% CI: −0.41 to −0.10; *p* = 0.001; *I*^2^: 76.5%) relative to children with ICPP (WMD: 0.15; 95% CI: −0.07–0.36; *p* = 0.174; *I*^2^: 70.0%) (Figure 3A). According to sample size, BMISDS was significantly decreased in trials with sample sizes ≥30 (WMD: −0.17; 95% CI: −0.31 to −0.03; *p* = 0.014; *I*^2^: 59.7%) relative to trials with sample sizes <30 (WMD: 0.05; 95% CI: −0.20–0.30; *p* = 0.692; *I*^2^: 83.9%) (Figure 3B). According to age, BMISDS was significantly decreased in trials involving children aged ≥8 years (WMD: −0.23; 95% CI: −0.40 to −0.05; *p* = 0.010; *I*^2^: 79.4%) relative to trials involving children aged <8 years (WMD: 0.08; 95% CI: −0.14–0.31; *p* = 0.481; *I*^2^: 77.2%) (Supplementary Figure 2).

Grouping trials separately by geographical region, treatment duration, and GnRHa usage status revealed no significant differences (Supplementary Figure 2).

### Cumulative and influential analyses

Cumulative analyses revealed no significant effect of the first published trial on subsequently published trials for the comparison of BMISDS between the intervention group and the control group (Supplementary Figure 3A). In influential analyses, the effect of single trials on overall effect-size estimates was nonsignificant (Supplementary Figure 3B).

### Meta-regression analyses

**Figure 4:**
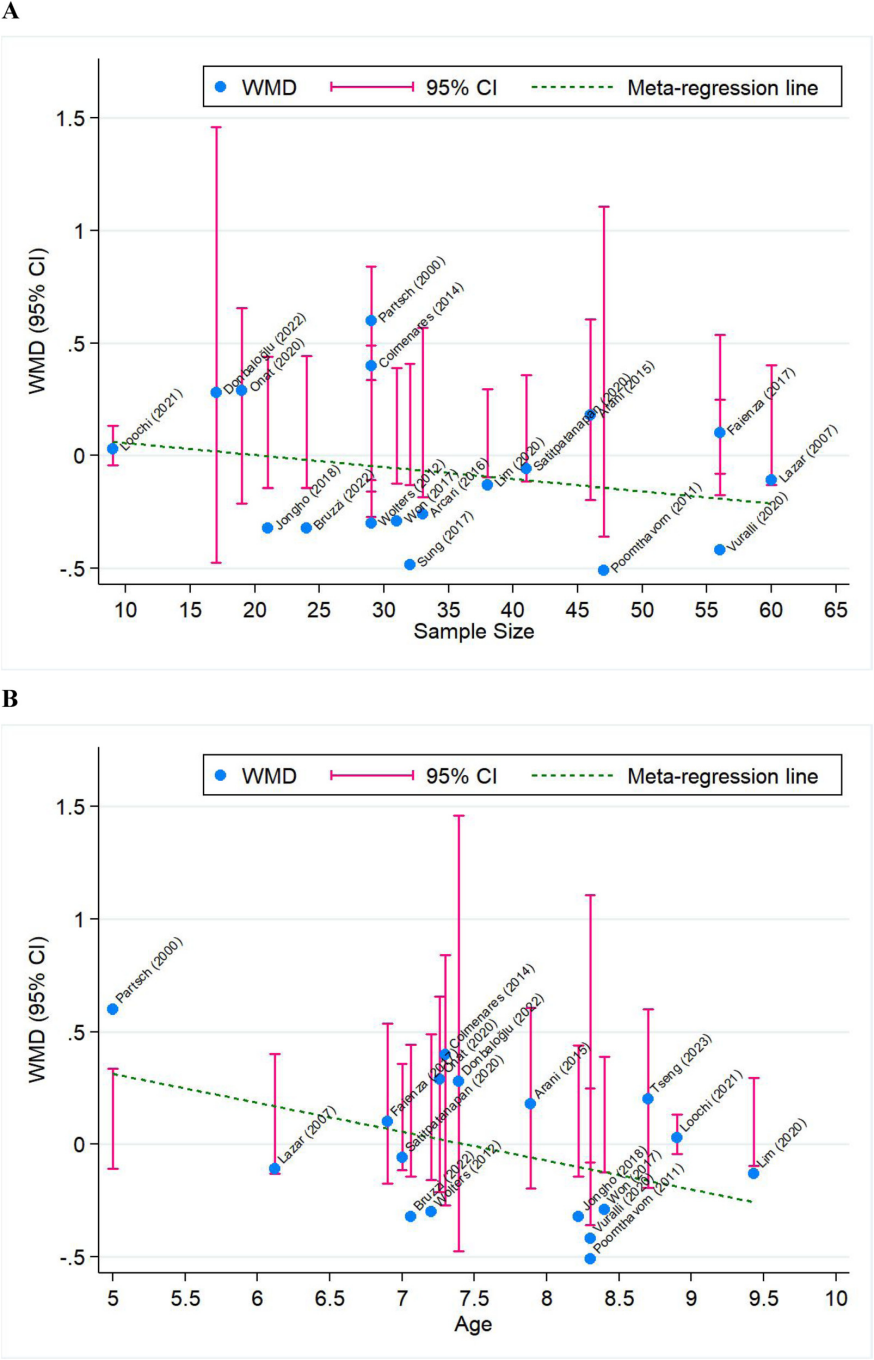
Meta-regression analysis for BMISDS by modeling case sample size **(A)** and averaged age **(B)**. WMD, weighted mean difference; 95% CI, 95% confidence interval. Blue solid circles represent effect-size estimates of individual trials, and pink vertical lines represent 95% CI of these effect-size estimates. The green dotted line represents the fitted regression line.

To explore further sources of between-trial heterogeneity, meta-regression analyses were performed by modeling case sample size and averaged age. BMISDS trended toward less significantly with the increase of case sample size (regression coefﬁcient, −0.004; *p* = 0.497) and averaged age (regression coefﬁcient, −0.118; *p* = 0.067), and progression exhibited a declining trend (Figure 4).

### Publication bias

**Figure 5:**
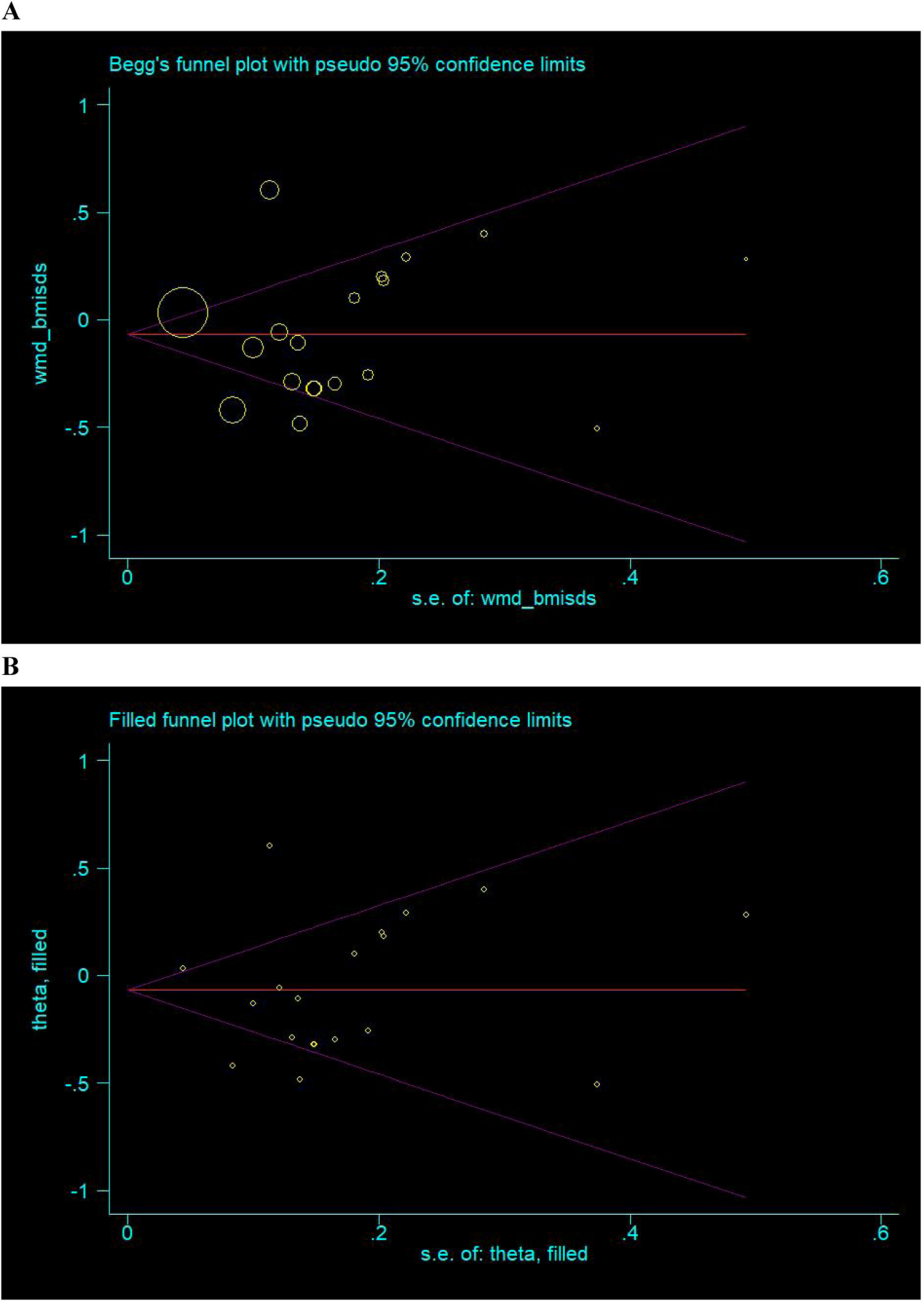
Begg's **(A)** and filled **(B)** funnel plots for the comparison of BMISDS. Hollow circles represent all eligible trials in this meta-analysis, and solid squares represent potentially missing trials required to achieve symmetry. WMD, weighted mean difference; 95% CI, 95% confidence interval.

Shown in Figure 5 are the Begg's and filled funnel plots for the comparison of BMISDS between the intervention group and the control group. The Begg's funnel plots seemed symmetrical (Figure 5A), and the Egger's tests indicated a low probability of publication bias (*p* = 0.801). In filled funnel plots, none missing trial was required to make the funnel plot symmetrical (Figure 5B).

## Discussion

In this meta-analysis, we aimed to investigate the metabolic changes in children with ICPP receiving GnRHa treatment. After pooling the results of 19 published researches, our analysis revealed that GnRHa treatment did not have a significant effect on BMISDS in children with ICPP, while it played a role in regulating HDL. To our knowledge, this is thus far the first meta-analysis that has assessed the effects of GnRHa treatment on metabolic changes in children with ICPP.

At present, the association between metabolic changes and GnRHa treatment in children with ICPP is not fully understood. Increased body weight was associated with precocious puberty in children (42). Patients with CPP have been found to exhibit high baseline BMISDS (43). Several studies found varied BMISDS during GnRHa therapy (17, 44). Other studies, however, showed no statistically significant changes during the treatment, consistent with the results of this meta-analysis (20, 21, 26). The effects of GnRHa treatment on adiposity are inconsistent. Several explanations are behind such divergence. The first possible explanation is between-trial heterogeneity. Age at baseline, BMI distribution, duration of treatment, and endpoint time may contribute to the different results of these studies. For example, BMISDS was reported to increase when receiving GnRHa treatment but decrease after cessation (39). As a result, the timing of BMISDS assessment may impact the results. In addition, most studies are retrospective and no randomized controlled trials are identified, rendering the results influenced by potential confounding factors. Moreover, some studies were single-arm, which only investigated the change of BMISDS before and after treatment in children with CPP, and were lacking parallel controls (17, 44). Besides BMI, lipid profiles were also meta-analyzed in our study. Our analysis revealed that GnRHa treatment might be related to higher HDL concentrations in children with ICPP, yet no statistical significance was found for TC, TG and LDL. *in vivo* experiment showed that the GnRH receptors were widely expressed in adipocytes. GnRHa can promote adipocyte maturation and increase the formation of lipid droplets in mature adipocytes (45). GnRHa treatment may influence the growth and metabolism of adipocyte and contribute to metabolic changes in children with CPP. However, there are too limited researches focusing on the changes of lipid profiles during GnRHa treatment in children with ICPP.

Our subgroup analysis revealed that children with overweight or obesity exhibited a significantly greater reduction in BMISDS following GnRHa administration compared to the overall study cohort. This suggests a differential treatment response in the children with overweight or obesity relative to children encompassing all weight categories. This observation aligns with previous reports indicating that normal-weight children tend to experience a more pronounced increase in BMISDS during GnRHa therapy compared to children with obesity (37, 39, 40, 46). It is noteworthy that BMISDS typically normalizes in adulthood (33). Several potential mechanisms may explain these differential effects: Firstly, Hormonal & Metabolic Factors: Arrigo et al. (44) proposed that the initial BMI increase often seen at GnRHa initiation may stem from pubertal hormonal changes. Furthermore, it has been proposed that GnRHa-induced suppression of the gonadal axis, leading to reduced estrogen production, may cause more significant adipose tissue redistribution or deposition in normal-weight children than in their overweight or obese counterparts. The pharmacological inhibition of the LH/FSH axis, which consequently reduces estrogen levels, is thought to be the underlying mechanism for the relatively greater adipose tissue accumulation observed in normal-weight children during treatment (47). Secondly, Psychological & Behavioral Factors: Psychological and behavioral aspects may also contribute to more substantial BMISDS changes in normal-weight children. Pich et al. (48) found that adolescent girls with obesity or overweight often demonstrate greater preoccupation with body shape and weight gain concerns compared to normal-weight peers. Consequently, they might engage more frequently in physical activity and weight monitoring, potentially aiding in better weight control during treatment. In contrast, the relatively slower growth rate observed in some normal-weight children compared to children with obesity (39) might also be a contributing factor to their more pronounced BMISDS increase. To further validate these observations and elucidate the underlying causes, future studies employing stratification by chronological age and precise BMI categories (specifically distinguishing between obese and normal-weight groups) are warranted.

Moreover, a declining trend in BMISDS with increasing sample sizes and increasing chronological ages at diagnosis was observed in our subgroup and meta-regression analyses, indicating that smaller sample sizes exhibited higher variability and the pharmacological effects of GnRHa varied across different age groups. Our further analyses provided insights into potential sources of heterogeneity, and the high *I*^2^ values suggested that additional unaccounted or unmeasured factors may also play a role. Future studies with standardized protocols and larger sample sizes are needed to elucidate the effects of these factors and account for heterogeneity. Nevertheless, it is noted that meta-regression analyses lack the methodological rigor of a properly designed clinical trial, which is intended to formally test the effects of these factors (49).

The results of our meta-analysis are inconsistent with that of previous studies. Luo et al. (50) reported the long-term effect of GnRHa treatment in children with CPP, including adult height improvement, BMI, menarche, polycystic ovary syndrome, and malignant diseases, showing that GnRHa treatment decreased the BMI of girls with CPP. However, only three articles were covered in this review. In our meta-analysis, 19 articles were included to explore BMISDS alternations. Additionally, we examine the effect of baseline weight and age on BMISDS in children with ICPP.

The benefit of GnRHa treatment is still debated (2). It has been demonstrated to play a role in preserving adult height potentials and postpone menarche to alleviate negative psychosocial stress. Our results focused on the metabolic results of GnRHa treatment and found that GnRHa treatment had little effect on the BMISDS of children with ICPP. However, children with overweight may exhibit a relatively slow increase in BMISDS during GnRHa treatment, and the difference is not statistically significant. Specialist assessment should be taken before the initiation of GnRHa to avoid overtreatment.

Despite the clear strengths of this meta-analysis including comprehensive search strategies, a wide range of related indexes, and comprehensive exploration of possible causes of between-trial heterogeneity, some limitations should be acknowledged. First, due to clinical ethics, no randomized controlled trials focusing on change of BMI during GnRHa treatment were identified through literature search. Second, limited numbers of trials are available for the indexes of lipid metabolism, and certain key metabolic parameters, particularly HbA1c and HOMA-IR, were not consistently reported across included studies. Third, the sample size of each trial was relatively small. Last but not the least, a substantial level of statistical heterogeneity was evident in our meta-analysis, possibly resulting from methodological heterogeneity of observational studies.

## Conclusions

Our meta-analytical findings indicate that GnRHa treatment did not appear to increase BMI and lipid metabolism levels in children with ICPP, irrespective of obesity status at the time of initiation therapy. Especially, we found that increased concentrations of HDL after GnRHa treatment. Clinicians should pay close attention to changes in BMI and lipid profiles during GnRHa treatment.

## Data Availability

The raw data supporting the conclusions of this article will be made available by the authors, without undue reservation.
